# Human Fascioliasis in Portugal: A Case Report

**DOI:** 10.7759/cureus.74527

**Published:** 2024-11-26

**Authors:** Sara Pereira, Paula Cerqueira, Sabina Azevedo, Bárbara Sousa, Selmira Faraldo

**Affiliations:** 1 Internal Medicine, Hospital Conde de Bertiandos, Unidade Local de Saúde do Alto Minho, Ponte de Lima, PRT; 2 Internal Medicine, Hospital Distrital de Santarém, Santarém, PRT

**Keywords:** fasciola hepatica, fascioliasis, liver abscess, parasitic liver disease, triclabendazole

## Abstract

Fascioliasis is a zoonotic disease that may affect humans as incidental hosts after the ingestion of contaminated water or aquatic plants. Despite the non-specificity of its signs and symptoms, a triad of abdominal pain, fever, and peripheral eosinophilia should increase suspicion. The diagnosis of fascioliasis can be particularly difficult in non-endemic countries. It presents a worldwide but heterogeneous distribution, according to several environmental and human activity-related factors. Few cases have been described in Portugal. The authors report the case of a 48-year-old man presenting with abdominal pain, fever, and nighttime sweating. The CT scan revealed multiple hepatic nodules, which were then confirmed as abscesses in the context of hepatic fascioliasis. We aim to alert for the persistence of a few autochthonous cases of human fascioliasis in Portugal, the need to maintain a high degree of suspicion for this disease despite its non-specific presentation, and the emergence of triclabendazole resistance.

## Introduction

Fascioliasis is a zoonotic disease that affects public health worldwide but is often neglected and underdiagnosed in developed countries. Its distribution depends on several factors, either environmental or human-related activities. In Portugal, there have been only 14 cases reported in the literature since 2000 [1-7].

The signs, symptoms, and analytical and imaging changes of fascioliasis can be nonspecific and mimic several hepatobiliary diseases. Its diagnosis can be challenging and requires a high degree of clinical suspicion. It is essential to collect a complete medical history, including the characterization of the area of ​​residence, work activity, hobbies, and eating habits. 

With this case report, the authors intend to exemplify the persistence of autochthonous cases of human fascioliasis in Portugal and the need to maintain a low threshold of suspicion for this disease, even in the face of non-specific manifestations. 

## Case presentation

The authors present the case of a 48-year-old man residing in a rural area in northern Portugal. He had a past medical history of obstructive sleep apnea syndrome but no other known illness. The patient denied the use of prescription or over-the-counter drugs. He was admitted to the emergency department for abdominal pain (diffuse, but worse in the right upper quadrant), diarrhea (watery diarrhea without blood or mucus), fever, and nighttime sweating that had been developing for a month. The patient denied previous similar episodes, respiratory or urinary complaints, weakness, anorexia, weight loss, vomiting, or skin changes. He also denied drug use, foreign travels, or recent infections. He worked as a cook in his own restaurant and sometimes practiced outdoor walks. The patient denied close contact with agricultural activities but reported sporadic consumption of locally produced raw watercress. His cohabitants were asymptomatic. There was no family history of malignancy. On physical examination, abdominal palpation was diffusely and mildly painful, with no identifiable masses or enlarged organs or signs of peritoneal irritation. The patient was hemodynamically stable, apyretic, and anicteric. He had no palpable adenopathies or other changes on physical examination.

**Figure 1:**
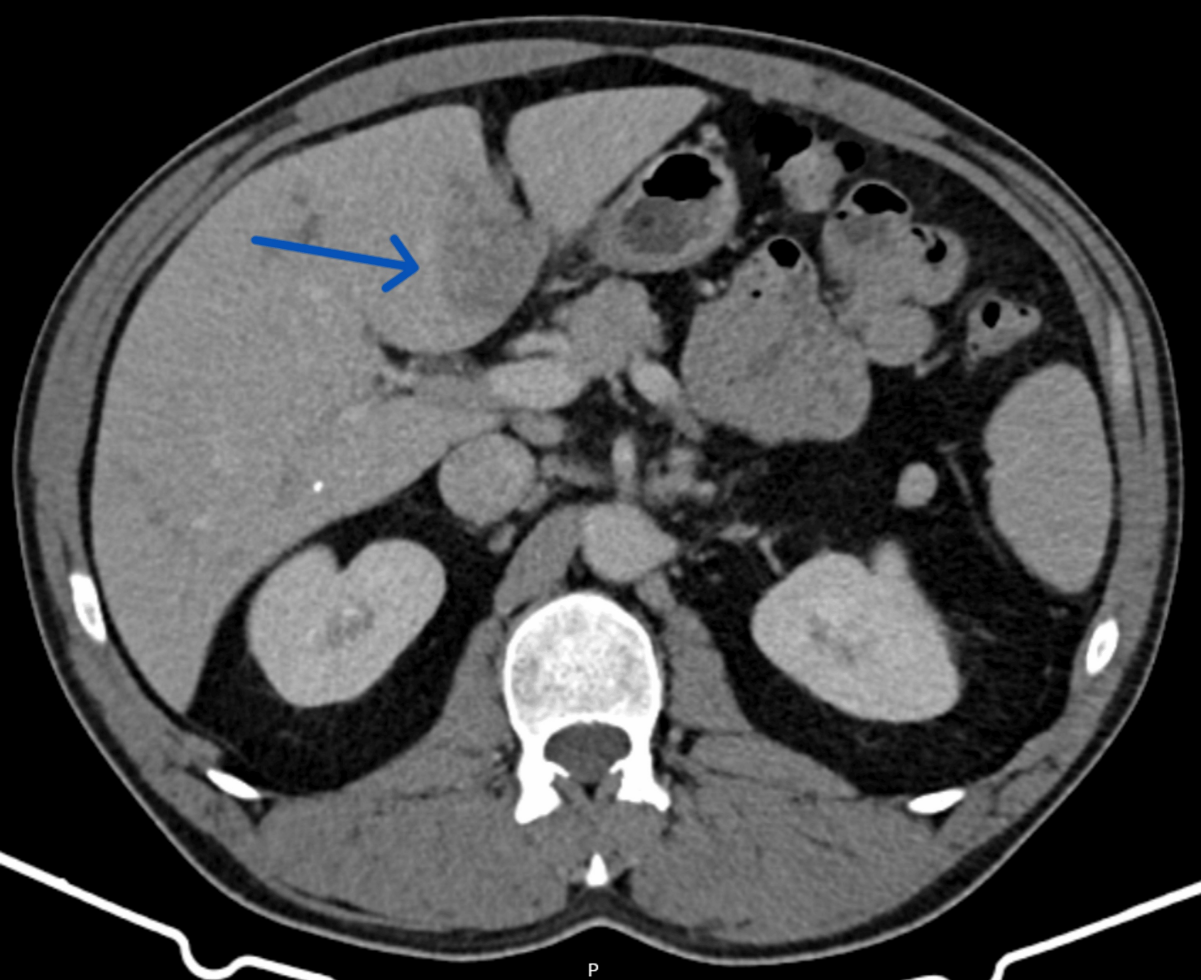
Abdominal CT scan performed on admission. The CT scan shows a heterogeneously enlarged liver with a craniocaudal diameter of 21 cm and scattered hypodense nodular lesions, some of which are confluent. In segment IVb, the largest lesion (arrow) has a diameter of 36 mm.

The bloodwork revealed eosinophilia (5.3x10^9^/L), cholestasis (alkaline phosphatase 316 U/L and gamma-glutamyl transferase 195 U/L), and elevated C-reactive protein (4.53 mg/dL). Platelet count (302,000/µL), serum bilirubin (total bilirubin 0.8 mg/dL), serum creatinine (0.7 mg/dL), aminotransferases (alanine aminotransferase 24 U/L and aspartate aminotransferase 29 U/L), and coagulation tests (international normalized ratio 1.0) were normal. The abdominal CT scan revealed a heterogeneously enlarged liver with scattered hypodense nodular lesions, some of which were confluent, with the largest one measuring 36 mm (Figure 1). There were also adenopathies in the hepatic hilum with a maximum diameter of 14 mm. There was no dilation of the intrahepatic or extrahepatic bile ducts. The patient was admitted to an internal medicine ward for a diagnostic workup.

**Figure 2:**
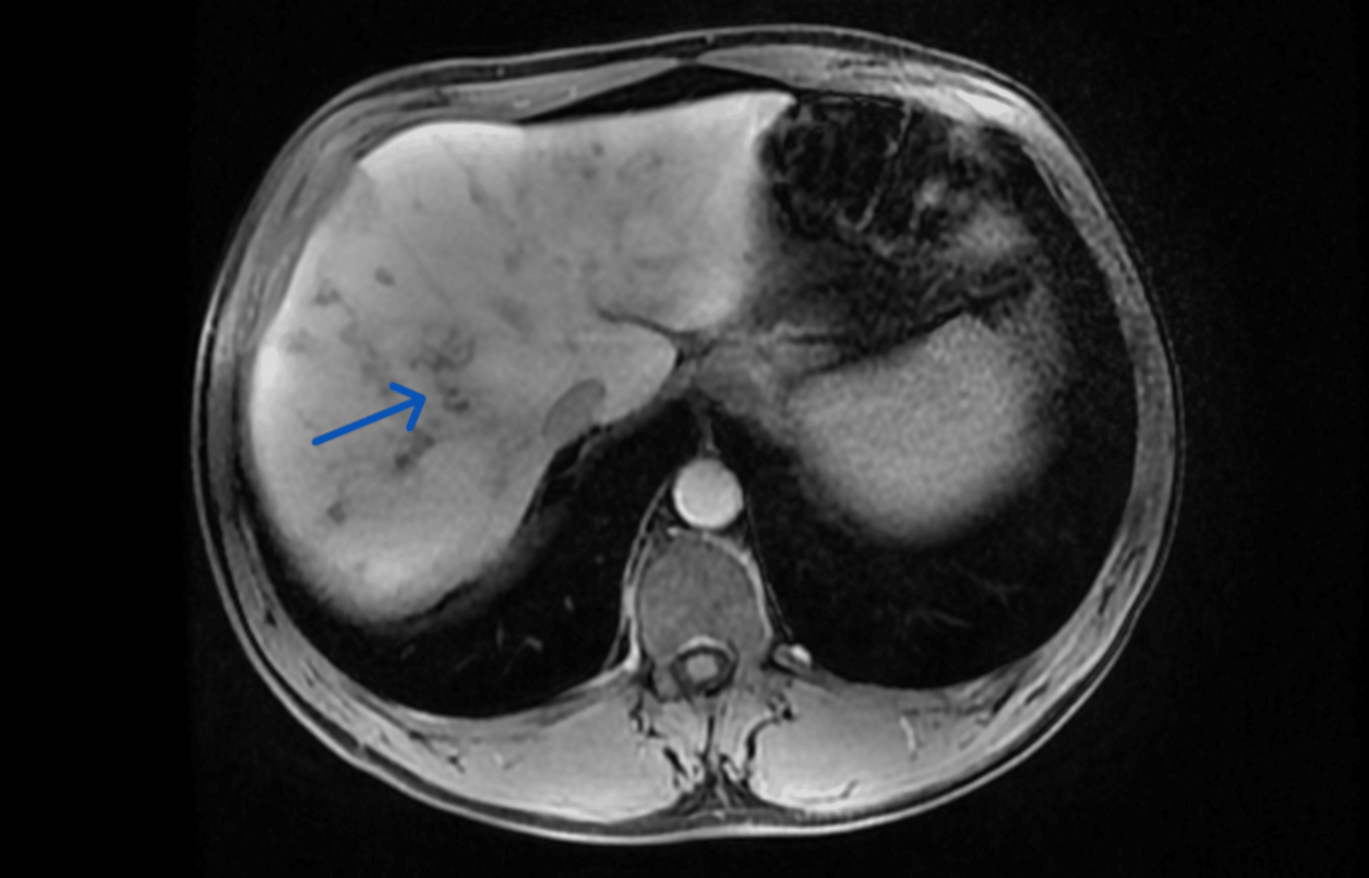
Abdominal MRI performed as part of the diagnostic workup. The abdominal MRI shows several poorly limited pseudonodular hepatic lesions, some of which are confluent, with subcapsular localization. Some lesions present a serpiginous pattern (arrow).

In Figure 2, the MRI scan shows several poorly limited pseudonodular hepatic lesions, some of which are confluent, with subcapsular localization, hypointense on T1, and hyperintense on T2 with peripheral enhancement in portal phase. Some lesions presented a serpiginous pattern. These findings were suggestive of infectious etiology and small liver abscesses. There was also slight dilation of the small bile ducts in segments VI and VII and peri-aortic, celiac, and portal adenopathies of likely reactive nature, with a maximum diameter of 14 mm. There were no signs of malignant disease.

Given the patient's report of sporadic consumption of locally produced raw watercress, a serology test for *Fasciola hepatica *was performed. The detection of antibodies for *Fasciola hepatica *was positive with a titer of 1/2560. The remaining microbiological studies were negative: serology tests for *Taenia solium, Entamoeba histolytica, Rickettsia conori, Schistosoma, Leishmania donovani, Echinococcus granulosis, Toxoplasma*, *Cytomegalovirus*, human immunodeficiency virus (HIV), hepatitis B virus (HBV), hepatitis C virus (HCV), Widal and Weil-Felix tests, bacteriological and parasitological examination of feces, and blood cultures. Immunological studies were also negative. The authors assumed that the infection by *F. hepatica* was transmitted by eating raw watercress. The patient was treated with a single dose of triclabendazole 10 mg/kg, with symptomatic and analytical improvement. The local Public Health Department was notified. The patient received recommendations regarding water and food consumption and was discharged home.

**Figure 3:**
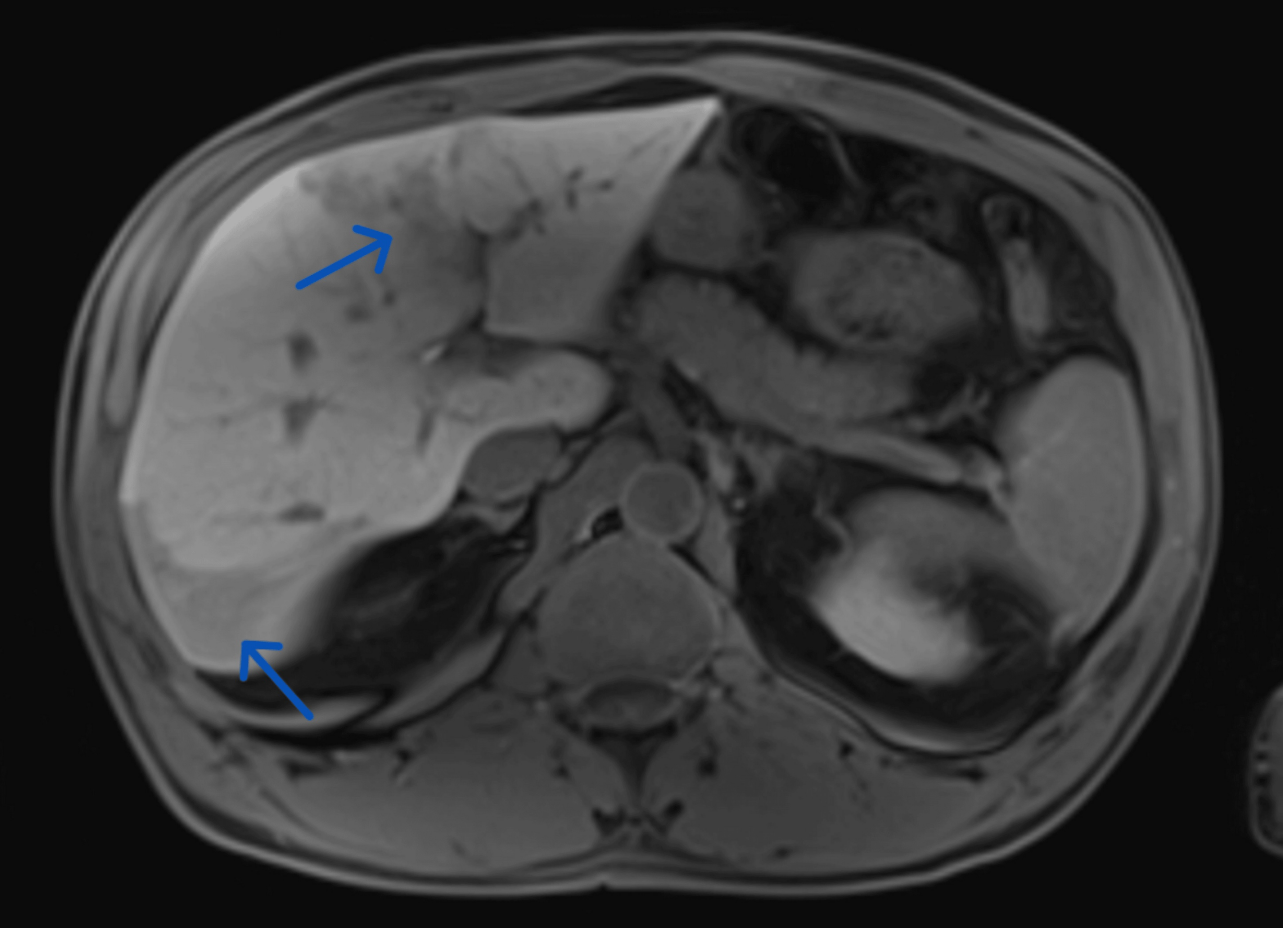
Abdominal MRI performed after first-line treatment with triclabendazole. The abdominal MRI shows an increase in the size of the pseudonodular hepatic lesions (arrows).

However, an MRI scan performed two months after treatment revealed worsening of the previously described abscessed lesions in size and number (Figure 3). The anti-*Fasciola hepatica* antibody test was repeated, remaining positive (with a titer of 1/640). After discussion with the local Infectious Diseases medical team, treatment was repeated with a course of high-dose triclabendazole (20 mg/kg/day) on two consecutive days. The patient did not report any side effects, remaining asymptomatic. Four months later, an MRI scan was performed, revealing significant improvement of the infectious lesions and discrete sequelae perfusion and fibrotic changes. 

## Discussion

Fascioliasis is a zoonosis with high prevalence among cattle, goats, and sheep, but it rarely affects human health. Human infection occurs by consumption of water or raw aquatic plants contaminated with the *metacercariae *form of *Fasciola hepatica* or *Fasciola gigantica,* trematodes with hepatobiliary tropism that are responsible for this disease.

Human fascioliasis is most prevalent in South America, the Middle East, and Southeast Asia [8], although its real global prevalence is not clear. In Europe, fascioliasis is more common in southern and eastern countries. There are insufficient Portuguese data on prevalence, with few cases described in the literature, mostly from patients living in rural areas of northern Portugal [1-7,9].

The prevalence of fascioliasis is characteristically higher in humid regions with intensive agricultural activity, but its geographic distribution depends on several dynamic factors and is constantly evolving. Environmental factors (such as climate and climate change) and factors related to human activities (agricultural and livestock management, housing and sanitary conditions, water conditions, local eating habits, travel, and migration patterns) contribute to complex interactions that will determine local patterns of infection and transmission of human fascioliasis [10-13]. Areas with a high prevalence of human fascioliasis may also differ from those with a high prevalence of ruminant infection because, besides animal infection, the availability of intermediary hosts and local dietary practices must be considered.

Fascioliasis presents with non-specific clinical manifestations, from asymptomatic forms to severe manifestations, with a significant impact on the quality of life of affected individuals. Its clinical manifestations can mimic several hepatobiliary conditions that must be considered as differential diagnoses, namely neoplastic disease. It usually presents itself in two phases: acute and chronic. The acute phase, in which the parasite migrates through the liver parenchyma causing inflammation and destruction, can manifest with fever, abdominal pain, nausea, hepatomegaly, pruritus, and peripheral eosinophilia. The acute phase usually lasts for six to 12 weeks. The chronic phase, in which the parasite settles in the bile ducts, can manifest with colicky abdominal pain, jaundice, hepatomegaly, weight loss, fatigue, and anemia. Complications such as cholangitis, cholecystitis, cholelithiasis, pancreatitis, bacterial superinfections, hepatocellular carcinoma, and cholangiocarcinoma may also occur [14]. The chronic phase can last for several years.

The most frequent analytical finding is peripheral eosinophilia, but hypergammaglobulinemia, anemia, elevated C-reactive protein, sedimentation rate, and elevated transaminases may also occur. Abdominal ultrasound may reveal hypoechoic subcapsular or peribiliary nodular lesions and thickening of the walls of the bile ducts. The most characteristic findings on CT and MRI scans are clustered hypodense and tortuous subcapsular lesions, with peripheral contrast enhancement and extension into the deeper parenchyma and portal regions [15]. On MRI, the lesions show T2 hypersignal. MRI shows signs of the evolutionary pattern of lesions resulting from the migration of the parasite through the liver parenchyma during the acute phase of the disease. It also allows a better characterization of complications, such as hemorrhagic lesions or abscesses.

Due to its clinical manifestations and imaging findings, fascioliasis may mimic neoplastic disease. Peripheral eosinophilia, especially in patients without risk factors for neoplasia, should prompt the consideration of parasitic infection, collection of an adequate clinical history, and investigation of the main microbiological agents. Imaging findings should be properly characterized with MRI before considering potentially unnecessary invasive procedures. The reported case reflects this approach.

Definitive diagnosis is made by serology, identification of the parasite in an endoscopic study or surgical specimen, or identification of eggs in feces. Serology tests, as performed in the reported case with identification of anti-*Fasciola hepatica* antibodies, and molecular biology by PCR have high sensitivity and specificity. The search for eggs in feces may be useful in the chronic phase of the disease, but not in the acute phase, because their elimination in feces depends on the existence of the parasite in the biliary tree.

The only drug proven to be effective and approved in the treatment of acute or chronic human fascioliasis caused by *F. hepatica or F. gigantica*, with estimated cure rates greater than 90%, is triclabendazole [16,17]. The dosage recommended for first-line treatment is not consensual. Therapeutic efficacy must be assessed from a symptomatic, analytical, and imaging perspective. In case of therapeutic failure, a second cycle of triclabendazole should be performed [17]. In the reported case, after first-line therapy, the patient showed clinical and analytical improvement but worsening of MRI-identified lesions. Treatment failure was assumed, and second-line therapy was performed with triclabendazole 20 mg/kg/day on two consecutive days. The patient remained asymptomatic, and imaging improvement was confirmed with a new MRI scan.

In order to increase its absorption in the intestine and avoid treatment failure, ingestion of a fatty meal before each triclabendazole dose is recommended. Treatment failure may also be caused by impaired hepatic metabolism in the presence of severe liver injury, which did not seem to be the case in our patient, or by fluke resistance to triclabendazole.

The exclusive use of triclabendazole over many years has resulted in an increasing rate of resistance and therapeutic failure in the last decade [16], with few cases of resistance described (including one Portuguese patient) [3]. The resistance mechanisms are not yet fully understood and are probably multifactorial [18]. Reports of resistance are worrying, given that triclabendazole is the only available highly effective treatment. Therefore, the need to study biomarkers of this resistance, different dosages and regimens of triclabendazole, and new drugs for the treatment of human fascioliasis increases.

The local socioeconomic and ecological context of each region must be considered in order to prevent fascioliasis. Several areas of intervention must be considered in order to reduce the risk of infection: water, sanitation, and hygiene; food practices; agricultural practices; veterinary public health; and population education on the subject [19]. In endemic areas, triclabendazole may be used as preventive therapy for the entire population. Currently, there is no vaccine for fascioliasis. Its development would be an important additional measure. Improvement of epidemiological surveillance systems could also contribute to increasing knowledge about the geographic distribution and transmission patterns of this disease. The complex, dynamic, and constantly evolving interactions between the environment, hosts, and human activities must be considered in order to prevent fasciolosis.

## Conclusions

Despite the implementation of measures to control animal and human fascioliasis, it still represents a public health issue in some regions. In Portugal, where its prevalence is significantly lower, diagnosing fascioliasis may be challenging. The reported case reinforces the importance of maintaining a high index of clinical suspicion when approaching a patient with peripheral eosinophilia and focal liver lesions, especially in the presence of suggestive epidemiology. Fascioliasis must be treated early and appropriately to avoid or minimize potentially serious complications. The possibility of therapeutic failure due to increasing rates of resistance to triclabendazole, the only proven and recommended agent in the treatment of fascioliasis, must be considered. Further studies are needed on the mechanisms of resistance and alternatives to triclabendazole. 

Complex and dynamic interactions between the environment, hosts, and human activities determine local infection patterns. To prevent and control fascioliasis, collaboration between sectors of human, animal, and environmental health is essential.
